# Pediatric bronchial Dieulafoy's disease: a biopsy-sparing bronchoscopic approach with selective bronchial artery embolization

**DOI:** 10.3389/fped.2026.1802778

**Published:** 2026-05-08

**Authors:** Weiqing Liu, Jiasi Zhou, Jia Guo, Tuanjie Wang, Yuping Xu, Shujun Li

**Affiliations:** 1 Children's Intensive Care Unit, The First Affiliated Hospital of Henan Medical University, Xinxiang, Henan, China; 2 Department of Transfusion Medicine, The First Affiliated Hospital of Henan Medical University, Xinxiang, Henan, China; 3 Department of Pediatric Hematology and Oncology, The First Affiliated Hospital of Henan Medical University, Xinxiang, Henan, China

**Keywords:** biopsy, bronchial artery embolization, bronchial Dieulafoy’s disease, bronchoscopy, children

## Abstract

**Background:**

Bronchial Dieulafoy's disease (BDD) is an exceedingly rare vascular malformation in children, characterized by aberrant submucosal arteries prone to life-threatening hemorrhage. The optimal diagnostic and therapeutic pathway remains undefined due to limited pediatric cases.

**Objective:**

To evaluate the feasibility and outcomes of a standardized pathway featuring biopsy-sparing bronchoscopy with typical endoscopic signs as triggers for selective bronchial artery embolization (BAE) in pediatric BDD.

**Methods:**

This single-center retrospective study analyzed consecutive children (<18 years) with hemoptysis who underwent selective angiography and BAE at the First Affiliated Hospital of Xinxiang Medical University from 2021 onwards. A predefined diagnostic pathway prioritizing airway safety, strict avoidance of biopsy at suspicious lesions, and early progression to BAE was implemented. Patients were classified as confirmed BDD (typical bronchoscopic findings without biopsy, angiographically confirmed) or highly suspected BDD (clinical phenotype consistent, angiographic abnormalities, hemostasis after BAE).

**Results:**

Three patients were included (median age 11 years). Two cases demonstrated typical bronchoscopic findings (submucosal elevation without pulsation) with strict biopsy avoidance and were classified as confirmed BDD. One patient bypassed bronchoscopy due to airway instability, classified as highly suspected BDD. All three patients in this cohort showed unilateral bronchial artery abnormalities on selective angiography, and computed tomography angiography (CTA) failed to identify definitive responsible vascular lesions in 100% (3/3) of cases. All patients achieved immediate hemostasis after superselective embolization (microspheres 300–500 μm ± coils). No major complications occurred. During follow-up (3–36 months), no recurrence was observed. Bronchoscopic localization matched angiographic laterality in 2/2 cases where bronchoscopy was performed.

**Conclusions:**

A standardized pathway emphasizing biopsy-sparing bronchoscopy and early BAE appears feasible and safe for pediatric BDD management, achieving favorable short-term outcomes even when computed tomography angiography (CTA) is non-localizing or bronchoscopy is bypassed due to clinical instability.

## Introduction

Bronchial Dieulafoy's disease (BDD), first described by Sweerts et al. in 1995, represents a rare vascular anomaly characterized by dysplastic arteries within the bronchial submucosa with potential for catastrophic hemorrhage (1, 2). The pathology involves abnormally dilated submucosal bronchial arteries that maintain their caliber without normal tapering, creating vessels prone to erosion or spontaneous rupture resulting in massive hemoptysis (3). While the condition has been well-documented in adults with over 100 cases reported, pediatric cases remain exceptionally rare, with fewer than 15 cases described in the literature (4, 5).

The pediatric presentation of BDD poses unique diagnostic and therapeutic challenges compared to adult cases. Children possess smaller blood volumes and limited compensatory reserves, rendering them particularly vulnerable to hemodynamic instability from acute blood loss (6). Additionally, the smaller airway dimensions in children increase the risk of asphyxiation from blood aspiration, while the technical limitations of pediatric bronchoscopy equipment complicate both diagnosis and intervention (7). The reported mortality rate in pediatric BDD exceeds that of adults, emphasizing the critical importance of rapid recognition and appropriate management (8).

Historically, the diagnosis of BDD relied heavily on bronchoscopic visualization of characteristic findings, including submucosal nodules with or without pulsation, the distinctive “white cap sign,” or active bleeding sites (9). However, the practice of obtaining tissue confirmation through biopsy has been associated with catastrophic hemorrhage, as these lesions represent high-pressure arterial malformations immediately beneath the mucosal surface (10). This absolute contraindication for biopsy in BDD is further reinforced by clinical evidence demonstrating that even attempted tissue sampling of these fragile submucosal arterial malformations can trigger life-threatening, even fatal, hemorrhage (11), leading to an evolution in diagnostic approaches with increasing emphasis on non-invasive imaging modalities and angiographic confirmation (11).

Computed tomography angiography (CTA) has emerged as a valuable tool in the evaluation of hemoptysis, offering non-invasive visualization of bronchial and non-bronchial systemic arteries (12). However, its diagnostic yield in pediatric BDD remains limited due to several factors: (1) the small caliber of pediatric vessels, (2) respiratory motion artifacts more pronounced in children, (3) radiation dose constraints necessitating lower contrast volumes and acquisition parameters, and (4) the intermittent nature of bleeding that may result in vessel spasm or thrombosis at the time of imaging (13).

Bronchial artery embolization (BAE) has revolutionized the management of massive hemoptysis, offering both diagnostic and therapeutic benefits with success rates exceeding 85% in most series (14). The technique has proven particularly valuable in pediatric patients who may be poor surgical candidates due to underlying comorbidities or acute instability (15). Recent advances in microcatheter technology and embolic materials have further improved the safety profile of BAE in children, though experience remains limited to specialized centers (16).

The development of standardized management protocols for rare diseases like pediatric BDD serves multiple critical functions. First, it reduces variability in clinical decision-making, particularly important when individual practitioners may encounter only single cases throughout their careers (17). Second, it establishes clear safety boundaries and decision points, minimizing the risk of iatrogenic complications from inappropriate interventions (18). Third, it facilitates multidisciplinary coordination between pediatric pulmonology, interventional radiology, intensive care, and thoracic surgery teams (19).

Despite these advances, significant knowledge gaps persist regarding optimal management strategies for pediatric BDD. The relative roles of bronchoscopy vs. early angiography, the safety of bronchoscopic evaluation without biopsy, and the long-term outcomes following BAE in children remain incompletely defined (20). Additionally, the management of patients presenting with hemodynamic instability or respiratory compromise, where traditional diagnostic algorithms may need modification, requires further clarification (21).

This study aimed to evaluate the feasibility and outcomes of a predefined diagnostic and therapeutic pathway for pediatric BDD that prioritizes airway safety through strict avoidance of biopsy at suspicious lesions, utilizes bronchoscopic findings as triggers for angiographic evaluation, and emphasizes early progression to BAE as both a diagnostic and therapeutic intervention. By analyzing our institutional experience with this standardized approach, we sought to contribute evidence supporting safe and effective management strategies for this rare but potentially fatal condition.

## Methods

### Study design and setting

This retrospective observational study was conducted at the First Affiliated Hospital of Xinxiang Medical University, a tertiary care center with dedicated pediatric pulmonology and interventional radiology services. The study protocol was approved by the institutional review board (EC-025-659), and written informed consent was obtained from all parents or legal guardians. The study period extended from January 2021 to December 2023, with follow-up data collected through June 2024.

### Patient selection

The study population consisted of pediatric patients presenting with hemoptysis who underwent evaluation according to our institutional BDD management protocol. Inclusion criteria were: (1) age less than 18 years at presentation, (2) presence of moderate to massive hemoptysis or recurrent hemoptysis episodes, and (3) completion of selective bronchial angiography with attempted BAE. Moderate to massive hemoptysis was defined as either 24-hour bleeding volume ≥8 mL/kg or single episode ≥4 mL/kg, hemodynamic instability requiring blood product transfusion, respiratory compromise with oxygen saturation <90% despite supplemental oxygen, or need for airway intervention including endotracheal intubation or bronchoscopic clearance. Recurrent hemoptysis was defined as two or more episodes requiring medical intervention within a 6-month period.

Exclusion criteria included: (1) confirmed alternative etiology sufficient to explain hemoptysis, including active tuberculosis, documented malignancy, diffuse bronchiectasis, foreign body aspiration, thoracic trauma, or established pulmonary vascular malformation, (2) uncorrected coagulopathy defined as platelet count <50 × 10⁹/L, international normalized ratio >1.5, or activated partial thromboplastin time >1.5 times upper limit of normal, (3) isolated blood-tinged sputum without frank hemoptysis, (4) incomplete medical records preventing assessment of key clinical variables, and (5) previous surgical lung resection for hemoptysis.

### Institutional management protocol

Prior to study initiation, a multidisciplinary team comprising pediatric pulmonology, interventional radiology, pediatric intensive care, anesthesiology, and thoracic surgery developed a standardized operating procedure (SOP) for suspected BDD management. The protocol emphasized several key principles: (1) prioritization of airway safety with early securing of definitive airway when indicated, (2) strict, absolute avoidance of biopsy or brush sampling at sites demonstrating characteristic BDD features (21), (3) utilization of bronchoscopic findings as triggers for progression to angiography rather than relying on CTA localization, and (4) early involvement of interventional radiology for definitive diagnosis and treatment.

The acute management algorithm began with rapid clinical stabilization includingincluding airway assessment (high-flow oxygen, bleeding-side-down positioning, continuous SpO_2_/EtCO_2_, aggressive suctioning of clots; if indicated, RSI with the largest feasible cuffed ETT to allow therapeutic bronchoscopy; lung isolation via selective mainstem intubation or a bronchial blocker), hemodynamic support (two large-bore IV/IO lines, 10–20 mL/kg isotonic crystalloid bolus, type & crossmatch, early blood products as needed; vasoactive infusion if shock—e.g., epinephrine 0.05–0.3 µg/kg/min or norepinephrine 0.05–0.2 µg/kg/min), and reversal of coagulopathy (stat CBC/PT/INR/aPTT/fibrinogen; targets INR < 1.5, platelets > 50 × 10⁹/L, fibrinogen > 150 mg/dL; vitamin K 0.3 mg/kg IV; FFP 10–15 mL/kg or PCC per protocol; platelets 10–15 mL/kg; cryoprecipitate 1–2 units/10 kg; adjunct tranexamic acid 10–15 mg/kg IV or 250–500 mg nebulized if not contraindicated). For patients with active bleeding or respiratory compromise, immediate airway protection was prioritized using the largest appropriate endotracheal tube to facilitate bronchoscopic intervention if needed. In cases of unilateral bleeding with contralateral lung contamination, lung isolation techniques including selective mainstem intubation or bronchial blocker placement were employed.

### Imaging evaluation

Chest radiography was performed as an initial screening examination in all patients, with particular attention to areas of consolidation, atelectasis, or other abnormalities suggesting bleeding source. Contrast-enhanced computed tomography of the chest was performed using a 64-slice scanner with standardized pediatric protocols adjusted for patient weight. CTA acquisition parameters included 1.25 mm slice thickness with 0.625 mm reconstruction intervals, tube voltage of 70–100 kVp based on patient size, and automatic tube current modulation. Intravenous contrast (iohexol 350 mgI/mL) was administered at 1.5–2.0 mL/kg via power injector with bolus tracking for optimal pulmonary and systemic arterial enhancement. While CTA served primarily as an exclusionary tool to identify alternative pathologies such as tumors, infections, or structural abnormalities, it was not considered a prerequisite for proceeding to angiography in clinically suspected BDD cases.

### Bronchoscopic evaluation

Flexible bronchoscopy was performed under general anesthesia with continuous cardiorespiratory monitoring when clinical stability permitted. The procedure utilized age-appropriate flexible bronchoscopes (Olympus BF-P290 for patients <20 kg, BF-P190 for patients ≥20 kg) introduced through endotracheal tubes sized to accommodate both ventilation and bronchoscope passage. Systematic examination of the tracheobronchial tree was performed with particular attention to identifying characteristic BDD features including: (1) smooth submucosal nodules or elevations, (2) presence of surface blanching suggestive of the “white cap sign,” (3) visible pulsation synchronous with cardiac cycle, and (4) contact bleeding with minimal manipulation.

When suspicious lesions were identified, strict protocol adherence mandated absolute, unconditional avoidance of biopsy, brush sampling, or aggressive lavage at these sites. Photographic documentation was obtained for all identified abnormalities. Limited saline lavage for airway clearance was permitted only in areas distant from suspicious lesions. For patients with ongoing active bleeding obscuring visualization, gentle suctioning and selective balloon tamponade were employed as temporizing measures. The primary objectives of bronchoscopy were lesion localization, exclusion of alternative pathologies, and guidance for subsequent angiographic evaluation rather than tissue diagnosis.

### Angiographic evaluation and embolization

Selective bronchial angiography was performed via transfemoral approach using 4-French or 5-French vascular sheaths depending on patient size. Initial aortography was performed to identify bronchial artery origins and exclude anomalous vessels. Selective catheterization of bilateral bronchial arteries was attempted using 4-French diagnostic catheters (Cobra or Simmons configurations) with subsequent superselective catheterization using 2.7-French microcatheters (Progreat, Terumo) when embolization was indicated.

Angiographic findings suggestive of BDD included: (1) bronchial artery hypertrophy with increased tortuosity, (2) areas of abnormal parenchymal staining or neovascularity, (3) arteriovenous shunting to pulmonary vessels, and (4) active contrast extravasation. When abnormal vessels were identified, careful evaluation for spinal artery branches was performed before embolization. Embolization was performed using polyvinyl alcohol particles (300–500 μm) as the primary embolic agent, with supplemental coil embolization for proximal vessel occlusion when indicated. The procedural endpoint was defined as cessation of abnormal parenchymal staining and reduction in flow through targeted vessels while maintaining some antegrade flow to prevent tissue necrosis.

### Diagnostic classification

Based on integrated clinical, bronchoscopic, and angiographic findings, patients were classified into two diagnostic categories: (1) Confirmed BDD: presence of characteristic bronchoscopic findings (submucosal elevation, white cap sign, or pulsation) without biopsy, with corresponding vascular abnormalities on selective angiography in anatomically concordant locations, and (2) Highly suspected/probable BDD: clinical presentation consistent with BDD (unilateral predominant hemoptysis without alternative etiology), angiographic demonstration of bronchial artery abnormalities (hypertrophy, tortuosity, neovascularity, or shunting), successful hemostasis following BAE, and absence of recurrence during follow-up period.

### Data collection and outcomes

Comprehensive data collection included demographic characteristics (age, sex, weight), clinical presentation features (hemoptysis volume, duration, associated symptoms)on admission, laboratory parameters (complete blood count, coagulation studies, inflammatory markers) on admission, imaging findings from all modalities, bronchoscopic findings when performed, angiographic findings and technical details, and procedural complications. Primary outcomes included: (1) immediate hemostasis defined as absence of frank hemoptysis within 24 h post-embolization, and (2) freedom from recurrent hemoptysis during follow-up. Secondary outcomes included: (1) major complications including neurological events, non-target embolization requiring intervention, procedural mortality, or need for emergent surgical intervention, (2) minor complications including access site complications, transient chest pain, or post-embolization syndrome, (3) hospital length of stay, (4) intensive care unit requirement and duration, and (5) need for blood product transfusion.

### Follow-up protocol

Post-discharge follow-up was scheduled at 2–4 weeks, 3 months, 6 months, and 12 months, with additional visits as clinically indicated. Follow-up evaluation included clinical assessment for hemoptysis recurrence, chest radiography at each visit, and repeat CTA at 6 months or earlier if clinically indicated. For patients with recurrent hemoptysis, repeat angiography with embolization was considered first-line management, with surgical resection reserved for embolization failures or technically unsuitable cases.

### Statistical analysis

Given the small sample size inherent to this rare disease, statistical analysis was primarily descriptive. Continuous variables were expressed as medians with ranges, while categorical variables were presented as frequencies and percentages. Concordance between bronchoscopic localization and angiographic findings was assessed in patients undergoing both procedures. Objective quantification of CTA diagnostic yield was performed by calculating the proportion of cases where definitive responsible vascular lesions were not identified. All analyses were performed using SPSS version 26.0 (IBM Corporation, Armonk, NY).

## Results

### Patient characteristics

During the study period, three pediatric patients meeting inclusion criteria were identified and treated according to the institutional BDD management protocol (Table 1). The cohort comprised two females and one male with ages ranging from 10 to 13 years (median 11 years). All three patients presented with massive hemoptysis, with single episode volumes ranging from 50 to 150 mL. No patient had significant past medical history or chronic respiratory conditions. The time from symptom onset to hospital presentation ranged from 5 h to 5 days, reflecting variation in initial bleeding severity and patterns.

**Table 1:** Patient characteristics and outcomes.

Parameter	Patient 1	Patient 2	Patient 3
Demographics			
Sex/age	Female/10 years	Male/13 years	Female/11 years
Clinical presentation			
Hemoptysis volume	50–100 mL	150 mL	150 mL
Presentation to admission (h)	48	120	5
Imaging			
CTA lateralization	Negative	Right (non-specific)	Right (non-specific)
CTA identification of definitive vascular lesion	No	No	No
Time to CTA (h)	3	1	1
Bronchoscopy			
Performed	Yes	Yes	No (unstable)
Time to bronchoscopy (h)	12	20	NA
Location of findings	RLL lateral basal segment (S9)	RLL lateral basal segment (S9)	NA
Pulsation present	No	No	NA
Biopsy performed	No	No	No
Angiography/Embolization			
Time to BAE (h)	24	31	16
Responsible vessel	Right BA (RLL S9)	Right BA (RLL)	Right BA (RLL)
Embolic material	PVA 300–500 μm	PVA 300–500 μm	PVA + coils
Outcomes			
Immediate hemostasis	Yes	Yes	Yes
Major complications	None	None	None
Hospital stay (days)	6	4	6
Follow-up (months)	36	6	3
Recurrence	No	No	No

CTA, computed tomography angiography; RLL, right lower lobe; BA, bronchial artery; PVA, polyvinyl alcohol; NA, not applicable.

### Clinical presentation and initial management

Patient 1, a 10-year-old female, presented with a 2-day history of recurrent hemoptysis without identifiable precipitating factors. Initial stabilization revealed hemodynamic stability, allowing for systematic diagnostic evaluation. Patient 2, a 13-year-old male, experienced intermittent hemoptysis over 5 days with progressive increase in bleeding volume, ultimately requiring intensive care monitoring and continuous positive airway pressure support due to respiratory compromise from blood aspiration. Patient 3, an 11-year-old female, presented with acute onset massive hemoptysis (150 mL) over 5 h with associated hypoxemia requiring immediate airway protection and mechanical ventilation.

### Imaging findings

**Figure 1:**
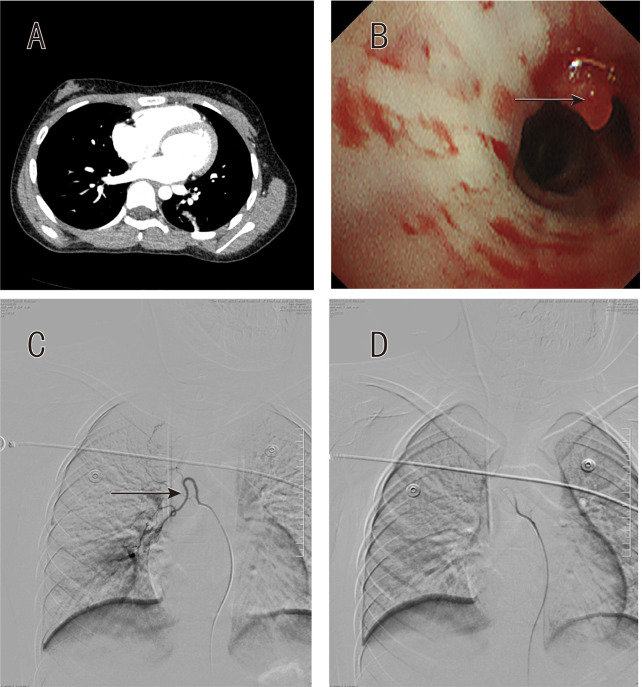
Patient 1 diagnostic and therapeutic sequence. **(A)** Computed tomography angiography showing no definitive vascular abnormality. **(B)** Bronchoscopic image demonstrating localized mucosal elevation in right lower lobe lateral basal segment without pulsation (arrow). **(C)** Selective right bronchial arteriography revealing tortuous vessels with chaotic branching pattern. **(D)** Post-embolization angiography confirming successful occlusion with absent distal flow.

**Figure 2:**
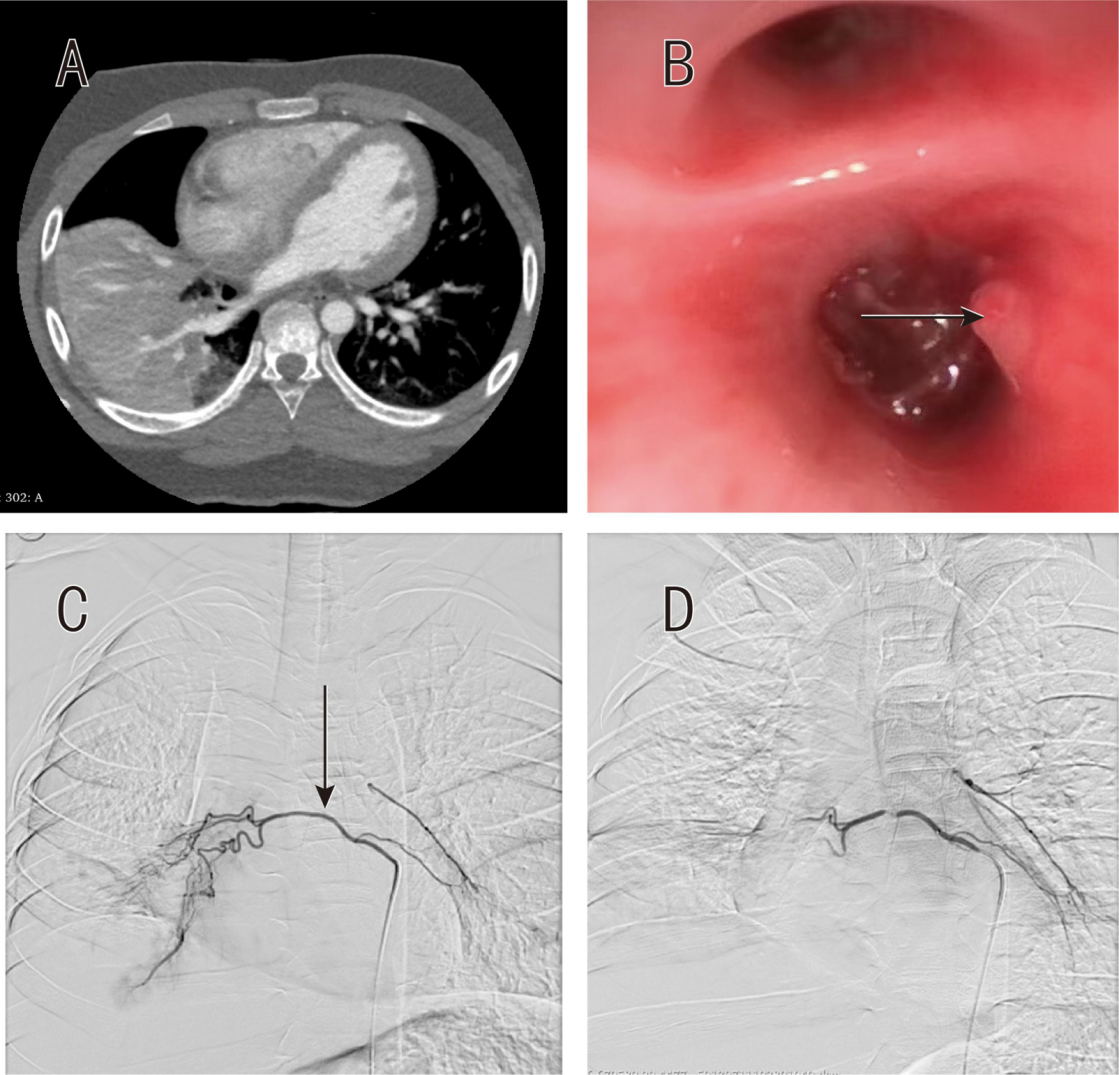
Patient 2 imaging findings. **(A)** CTA demonstrating right-sided ground-glass opacification without clear vascular etiology. **(B)** Bronchoscopic view after clot evacuation showing mucosal elevation at right lower lobe bifurcation (arrow). **(C)** Bronchial arteriography revealing hypertrophied right bronchial artery with early pulmonary venous opacification (arrowhead). **(D)** Post-embolization image showing successful elimination of arteriovenous shunting.

**Figure 3:**
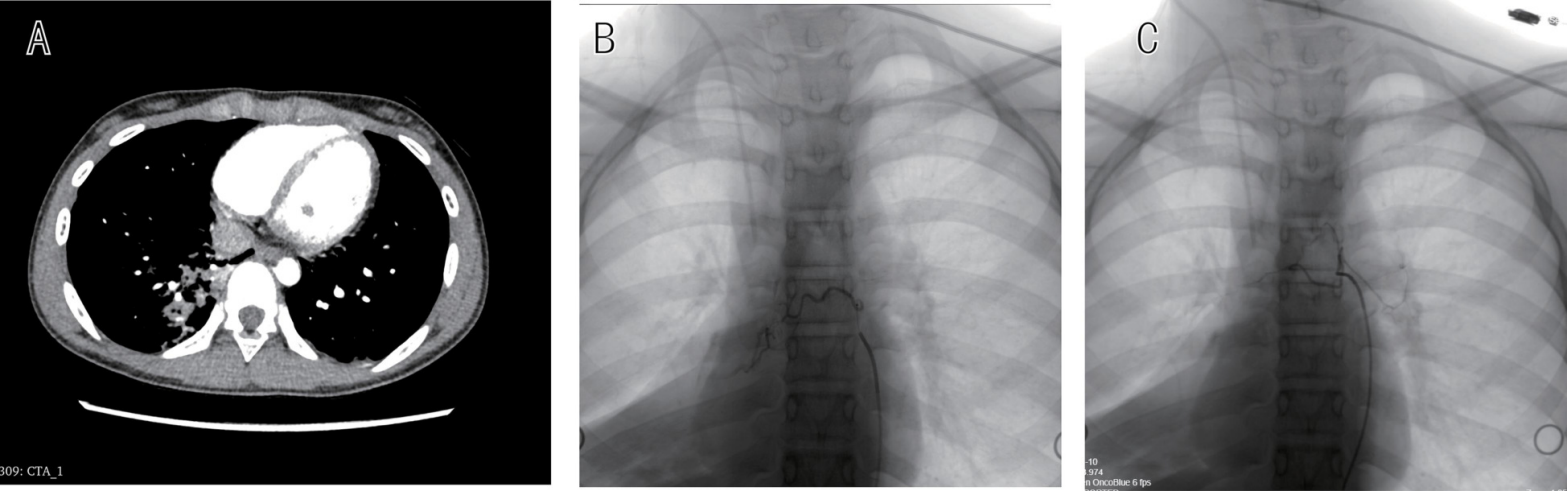
Patient 3 angiographic management without bronchoscopy. **(A)** CTA showing extensive right-sided hemorrhagic consolidation. **(B)** Urgent bronchial arteriography demonstrating right bronchial artery hypertrophy with abnormal parenchymal staining. **(C)** Post-embolization angiography confirming marked flow reduction in target vessel.

Chest radiography demonstrated unilateral opacification suggestive of alveolar hemorrhage in all three cases, with right-sided predominance noted universally. CTA was performed within 1–3 h of admission in all three patients, and definitive responsible vascular lesions were not identified in 100% (3/3) of the cohort (Table 1). Patient 1 showed no vascular abnormalities on CTA (Figure 1A), while Patient 2 demonstrated extensive ground-glass opacification consistent with alveolar hemorrhage but without clear vascular etiology (Figure 2A). Patient 3 similarly showed extensive right-sided hemorrhagic consolidation without visualization of abnormal systemic arteries (Figure 3A). The non-contributory nature of CTA findings necessitated progression to invasive angiographic evaluation in all cases.

### Bronchoscopic findings

Bronchoscopy was successfully performed in two patients (Patients 1 and 2) following initial stabilization. Both procedures identified characteristic findings in the right lower lobe. Patient 1 demonstrated a localized mucosal elevation at the right lower lobe lateral basal segment (RLL S9) without visible pulsation (Figure 1B). Patient 2, following bronchoscopic clot evacuation from the right main bronchus, revealed mucosal elevation at the bifurcation of the right lower lobe lateral basal segment (RLL S9), similarly without pulsation (Figure 2B). In both cases, strict adherence to the biopsy-avoidance protocol was maintained despite the absence of active bleeding at the time of examination. Patient 3 did not undergo bronchoscopy due to persistent hemodynamic instability and ongoing active hemorrhage requiring immediate progression to angiography.

### Angiographic findings and intervention

Selective bronchial angiography was performed at 16–31 h after admission (median 24 h), with earlier intervention in Patient 3 due to clinical instability (Table 1). All three patients demonstrated unilateral right bronchial artery abnormalities. Patient 1 showed a tortuous right bronchial artery with chaotic branching pattern supplying the right lower lobe lateral basal segment (RLL S9) (Figure 1C). Patient 2 revealed a common bronchial trunk with a hypoplastic left bronchial artery and hypertrophied right bronchial artery demonstrating increased branching, clustering of vessels, and early pulmonary venous opacification suggestive of arteriovenous shunting in the right lower lobe (RLL) (Figure 2C). Patient 3 demonstrated right bronchial artery hypertrophy with increased tortuosity and abnormal parenchymal staining in the right lower lobe (RLL) (Figure 3B).

Superselective embolization was technically successful in all cases using 300–500 μm polyvinyl alcohol particles. Patients 1 and 2 received particle embolization alone, while Patient 3 required supplemental coil placement for proximal vessel occlusion. Post-embolization angiography confirmed successful occlusion with absent distal flow in Patient 1 (Figure 1D), elimination of arteriovenous shunting in Patient 2 (Figure 2D), and marked flow reduction in Patient 3 (Figure 3C). No evidence of spinal artery compromise was identified in any patient.

### Immediate outcomes

All three patients achieved immediate hemostasis within 24 h of embolization (Table 1). No major complications occurred, including absence of neurological deficits, non-target embolization, or need for surgical intervention. Minor complications were limited to transient chest pain in Patient 2, managed with analgesics. Hospital length of stay ranged from 4 to 6 days (median 6 days). Patients 2 and 3 required initial intensive care monitoring for 24–48 h before transfer to general pediatric wards. No patient required blood product transfusion.

### Follow-up outcomes

Follow-up duration ranged from 3 to 36 months. Patient 1, with the longest follow-up of 36 months, remained free from hemoptysis recurrence with normal activity levels and no respiratory limitations. Patient 2, followed for 6 months, similarly demonstrated no bleeding recurrence. Patient 3, with 3 months of follow-up at study conclusion, maintained clinical stability without hemoptysis. Serial chest radiography showed resolution of previous hemorrhagic consolidation in all patients. Follow-up CTA performed at 6 months in Patients 1 and 2 demonstrated no evidence of revascularization or collateral formation.

### Concordance of findings

In the two patients undergoing both bronchoscopy and angiography, complete concordance was observed between bronchoscopic localization and angiographic laterality (2/2, 100%). Both patients with right lower lobe bronchoscopic findings demonstrated corresponding right bronchial artery abnormalities, supporting the diagnostic value of careful bronchoscopic examination even without tissue confirmation.

## Discussion

This study presents a systematic approach to managing pediatric bronchial Dieulafoy's disease through implementation of a standardized protocol emphasizing biopsy-sparing bronchoscopy and early progression to selective BAE. Our experience with three consecutive cases demonstrates the feasibility and safety of this approach, with all patients achieving immediate hemostasis and maintaining freedom from recurrence during follow-up. These findings contribute valuable evidence to the limited pediatric literature on BDD management, where previous reports have consisted primarily of isolated case descriptions without systematic protocol evaluation (22). Notably, this standardized pathway aligns with the experience of Lai et al. (4) in their two pediatric BDD cases managed with BAE, who also reported successful immediate hemostasis and short-term freedom from recurrence without major complications—further validating the efficacy of early BAE as a core component of pediatric BDD management. A systematic review of the limited pediatric BDD literature [encompassing fewer than 15 reported cases to date (4, 5)] confirms that BAE has emerged as the first-line therapeutic intervention for this condition, with consistent rates of immediate hemostasis and low complication rates in specialized centers (4, 5, 15).

The pathophysiology of BDD involves aberrant submucosal arteries that maintain abnormal caliber without normal tapering, creating high-pressure vascular channels immediately beneath the bronchial mucosa (23). This anatomical configuration explains both the propensity for spontaneous hemorrhage and the catastrophic bleeding risk associated with biopsy attempts. Recent histopathological studies have demonstrated that these vessels often lack the normal medial smooth muscle layer, potentially contributing to their fragility and tendency toward rupture (24). Understanding the underlying pathology confirms that biopsy is absolutely contraindicated in BDD. As Malegaonkar (25) stressed, biopsy of these superficial, high-pressure submucosal arterial malformations—lacking protective tissue between the artery and bronchial lumen—is a critical clinical error that may trigger fatal hemorrhage. This firmly justifies our protocol's strict biopsy avoidance, eliminating iatrogenic bleeding risk. This approach departs fundamentally from conventional bronchoscopic practice that relies on tissue diagnosis, and is validated by mounting evidence of lethal hemorrhage associated with BDD biopsy (26). The characteristic bronchoscopic findings of smooth submucosal elevation, with or without the white cap sign, provide sufficient diagnostic information when combined with appropriate clinical context and angiographic confirmation. Our experience demonstrates that careful photodocumentation of these findings, without tissue sampling, maintains diagnostic accuracy while eliminating iatrogenic bleeding risk.

The limited diagnostic yield of CTA in our cohort—with definitive responsible vascular lesions not identified in 100% (3/3) of patients—aligns with recent pediatric imaging studies highlighting the technical challenges of visualizing small-caliber bronchial arteries in children (26). Several factors contribute to this limitation: pediatric bronchial arteries typically measure 1–2 mm in diameter, respiratory motion is more pronounced in anxious or dyspneic children, and radiation dose constraints necessitate lower contrast volumes and reduced acquisition parameters (27). Furthermore, the intermittent nature of bleeding in BDD may result in vasospasm or thrombosis at the time of imaging, further reducing visualization of culprit vessels. These limitations support our protocol's approach of using CTA primarily as an exclusionary tool rather than requiring positive findings before proceeding to angiography.

The role of endobronchial ultrasound (EBUS) in BDD diagnosis represents an emerging area of interest. Recent adult studies have demonstrated that radial EBUS can differentiate vascular from non-vascular submucosal lesions, potentially providing additional confirmation of BDD without biopsy risk (28). Color Doppler evaluation during EBUS can demonstrate arterial flow patterns within suspicious lesions, offering real-time hemodynamic assessment. While our current protocol did not incorporate EBUS due to equipment limitations, future iterations should consider this modality as an adjunctive diagnostic tool, particularly as pediatric-specific EBUS equipment becomes more widely available (29).

Selective bronchial angiography serves dual diagnostic and therapeutic purposes in BDD management. The angiographic findings in our cohort, including arterial hypertrophy, abnormal branching patterns, and arteriovenous shunting, are consistent with previously described features of BDD (30). The identification of early pulmonary venous opacification in Patient 2 suggests the presence of bronchopulmonary shunting, a finding that may predict higher recurrence risk and warrant closer follow-up—a finding also noted in Lai et al.'s (4) pediatric BDD cases, where angiographic arteriovenous shunting was identified in one patient and managed successfully with superselective BAE. The universal right-sided predominance in our series, while potentially coincidental given the small sample size, aligns with anatomical studies suggesting that the right bronchial circulation may be more susceptible to developmental anomalies (31).

The technical aspects of pediatric BAE require careful consideration of age-specific anatomical and physiological factors. The smaller vessel caliber in children necessitates meticulous catheter selection and manipulation to achieve stable position for embolization (32). Our choice of 300–500 μm particles as the primary embolic agent reflects a balance between achieving effective occlusion and minimizing tissue ischemia risk. This particle size is large enough to avoid capillary bed penetration while small enough to reach the peripheral arterial branches responsible for hemorrhage. The supplemental use of coils in Patient 3 illustrates the occasional need for proximal vessel control in cases with high-flow states or large feeding arteries (33)—a technical modification also utilized in complex pediatric BDD cases reported by Lai et al. (4) for achieving durable vascular occlusion.

The absence of major complications in our series, particularly spinal cord ischemia, reflects adherence to established safety principles for bronchial embolization. Systematic evaluation for spinal artery branches, including the artery of Adamkiewicz, is mandatory before embolization (34). The use of superselective catheterization techniques allows precise embolic delivery while minimizing non-target embolization risk. Post-procedural monitoring for signs of spinal cord compromise, including serial neurological examinations and assessment of lower extremity function, should continue for at least 24–48 h given the potential for delayed presentation of ischemic complications.

Long-term outcomes following BAE for pediatric BDD remain incompletely characterized due to limited case numbers and follow-up duration. Adult series report recurrence rates ranging from 10% to 30%, with higher rates observed in patients with bilateral disease, incomplete initial embolization, or underlying inflammatory conditions (35). Our cohort's freedom from recurrence during follow-up periods extending to 36 months is encouraging but requires longer-term validation—consistent with the short-to-medium term follow-up results of Lai et al. (4), who reported no recurrence in their two pediatric BDD patients at 6 and 12 months post-BAE. The potential for recanalization or collateral formation necessitates continued surveillance, with repeat CTA at 6–12 month intervals during the first 2 years post-embolization appearing reasonable based on adult experience.

The successful management of Patient 3 without bronchoscopy highlights an important protocol flexibility for clinically unstable patients. While bronchoscopic localization provides valuable anatomical information, delaying definitive therapy in unstable patients to perform bronchoscopy may increase morbidity and mortality risk. Our protocol's allowance for direct progression to angiography in such cases, guided by clinical judgment and imaging findings, reflects real-world practice requirements where patient safety takes precedence over diagnostic completeness. This approach aligns with recent recommendations emphasizing individualized decision-making in pediatric interventional procedures (36).

Multidisciplinary collaboration emerged as a critical success factor in our protocol implementation. The involvement of pediatric pulmonology, interventional radiology, intensive care, and anesthesiology from initial presentation through follow-up ensured comprehensive patient care and optimal resource utilization. Regular multidisciplinary meetings to review cases and refine protocols facilitated continuous quality improvement. This team-based approach is particularly important for rare diseases like BDD, where individual practitioners may have limited personal experience and benefit from collective expertise (37).

Several limitations of our study warrant consideration. The small sample size, while consistent with the rarity of pediatric BDD, limits statistical power and generalizability. The retrospective design introduces potential selection and information bias, though prospective studies of such rare conditions present feasibility challenges. The single-center nature of our experience may not reflect practice patterns or outcomes at other institutions with different resources or expertise. Additionally, our follow-up duration, while extending to 36 months in one case, remains insufficient to fully characterize long-term recurrence risk and late complications.

Future research directions should focus on several key areas. Multicenter collaborative registries could aggregate sufficient case numbers to better define optimal management strategies and identify predictors of treatment success—a critical next step given the paucity of pediatric BDD cases and the need to validate standardized protocols across different clinical settings, building on the single-center experiences of our study and Lai et al. (4). Prospective validation of diagnostic criteria, including the role of advanced imaging modalities like EBUS and optical coherence tomography, could refine diagnostic algorithms. Investigation of novel embolic agents, including liquid embolics and drug-eluting particles, may improve treatment durability. Finally, development of pediatric-specific quality metrics and outcome measures would facilitate standardized reporting and comparison across centers.

The implications of our findings extend beyond BDD management to broader considerations in pediatric interventional pulmonology. The successful implementation of a standardized protocol for a rare disease demonstrates the value of systematic approaches in reducing practice variability and improving outcomes. The emphasis on avoiding potentially harmful diagnostic procedures when safer alternatives exist reflects evolving concepts of diagnostic stewardship in pediatric medicine. Finally, the integration of interventional radiology techniques into pediatric pulmonary disease management highlights the importance of developing specialized expertise and maintaining appropriate credentialing standards.

## Conclusions

This study demonstrates the feasibility and safety of a standardized management pathway for pediatric bronchial Dieulafoy's disease that prioritizes patient safety through strict avoidance of biopsy at suspicious lesions while facilitating early definitive therapy through selective bronchial artery embolization. All three patients in our cohort achieved immediate hemostasis without major complications and remained free from recurrence during follow-up. The protocol's flexibility, allowing for bypassing bronchoscopy in unstable patients while maintaining diagnostic accuracy through angiographic confirmation, addresses real-world clinical challenges in managing this rare but potentially fatal condition. CTA failed to identify definitive responsible vascular lesions in 100% (3/3) of our patients, underscoring the limitations of this imaging modality in pediatric BDD. While our findings are encouraging, the small sample size and single-center design necessitate multicenter validation. Future collaborative efforts should focus on prospective registry development, refinement of diagnostic criteria, and establishment of evidence-based follow-up protocols to optimize long-term outcomes in pediatric BDD.

## Data Availability

The original contributions presented in the study are included in the article/Supplementary Material, further inquiries can be directed to the corresponding author.
